# Supplementary material for: Factors associated with viral load suppression among orphans and vulnerable children and adolescents living with HIV in Kenya
Source: PLOS Glob Public Health. 2023 Mar 23;3(3):e0000794. doi: 10.1371/journal.pgph.0000794 (PMC10035747; doi:10.1371/journal.pgph.0000794)
Supplement: Attachment — Submitted filename: Response to Reviewers Comments_February 10th 2023_HNJDRB.pdf [file pgph.0000794.s002.pdf]

Response to Editor’s and Reviewers’ Comments

Factors associated with viral load suppression among orphans and vulnerable children and adolescents living with HIV in Kenya (Manuscript ID: PGPH-D-22-00954)

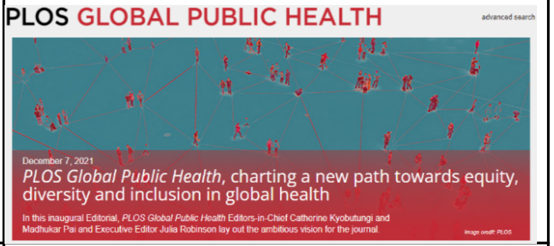

February 12th, 2023

Dear Dr Claudia P. Cortes,

Thank you for inviting us to submit a revised manuscript. We are grateful to the editor and reviewers for their insightful comments and suggestions that, we believe, strengthen this manuscript. My team has taken time to work on the manuscript again. Please, allow us to present detailed responses to reviewers’ comments in Table format:

Sincerely,

Brian Onyango.

==

Response to Review Comments to the Author

Response to Reviewer #2 Comments

Reviewer #2 Comments		Author’s Responses
1	Consider re-casting the results and conclusion sections of the abstract to read as follows (detailed below): Results: Determinants of VL suppression included child’s education status (aOR = 1.33; 95% CI: 1.07, 1.65), membership of a psychosocial support group (aOR = 1.258; 95% CI: 1.15, 1.38), and membership of	The team appreciates the feedback provided in relation to the abstract section. We have adopted the suggestion offered to re-cast the results and conclusion sections of the abstract. Additionally, the information related to the proportions of sites that were rural and urban that was left blank has now been added (see track changes).

	a voluntary savings and lending association (VSLA) (aOR = 1.226; 95% CI: 1.129, 1.33). In addition, child's sex (aOR=0.88; 95% CI: 0.83, 0.94), caregiver sex (aOR=0.909; 95% CI: 0.839, 0.997) and "high" status for caregiver household vulnerability (aOR = 0.81; 95% CI: 0.71, 0.924), had an inverse relationship with VL suppression.<br>Conclusion: CALHIV characteristics including child's sex, child's education status (whether currently active in school or inactive) and child's membership in a psychosocial support group were key determinants of VL suppression. Similarly, caregiver sex and membership in a voluntary savings and lending association also influence VL suppression.<br><br>Additionally, the proportions of sites that were rural and urban are currently missing (underlined in red in the attached PDF)<br>-to be included	

Response to Reviewer #3 Comments

Reviewer #3 Comments		Author's Responses
1	The impact of the constructs examined in the analysis are not well developed. The authors note that "Several reasons cited for poor adherence to ART include financial instability, poverty and food insecurity, limited access to medical resources, and costs of transportation. Good adherence and retention are noted to be necessary requirements for achieving viral suppression. Adolescents are particularly vulnerable to household poverty, yet adherence to treatment regimens requires a level of economic stability that many poor youths in Africa do not have/experience [28][29]." However, do not go into detail as to how this may impact viral suppression. What is the conceptual	Thanks again to the reviewer for this insightful comment. The underpinnings of the importance of economic stability being protective and helpful are described in literature. Several publications serve to underscore our inferences. The narratives touching on this issue have been revised to make it more explicit in the manuscript. Additionally, the theoretical framework underpinning the study has also been introduced.

	framework underpinning the assumption that economic stability might be protective and helpful? There are multiple frameworks that could be described to set the article up in the introduction section better.	
2	Also, there are other factors that are examined that relate potentially to structural barriers to care - ie, distance to the facility, adequate training, that are included in the analysis but not introduced from a theoretical perspective. Why might these be helpful in exploring?	We thank the reviewer for this comment and have included an additional reference in the background that cites influence of structural barriers to care. Again the theoretical framework underpinning the study has identified the issue of distance to health facilities as a contributing factor to ART adherence and therefore viral load suppression.
3	The authors provide information in the results that should be included in the methods. For example the description and handling of missing data should be moved to the methods section.	We thank the reviewer for this comment and have included and therefore, the model section narrative including the discussion related to the missing data has been moved to the method section as advised.
4	The authors describe that the model was initially not stable and did not converge. "Owing to model non-convergence, two independent variables among the caregiver characteristics were eliminated from the multivariable model: "Basic community HIV management training" and "case management training for case worker." These variables were removed. Were these thought to be correlated? If so, please provide a correlation coefficient. If not, the authors need to justify why these variables were removed. Currently, it looks like it was randomly determined which is not a thoughtful approach.	The variables were eliminated due to data multicollinearity. This was noted between these three sets of training i.e Case Management Training, PSS Training and basic community HIV management training. All for case workers.
5	The focus of the article is only included in 2 sentences in the discussion. "Voluntary Savings and Lending Associations (VLSA), and elevated odds of VL suppression in our study is similar to findings from an earlier study in Soweto, South Africa. [21]. In this study, the intervention involved CBO empowering families of OVC by providing income generating activities. Similarly, through provision of opportunities for saving and lending,	Again, we thank the reviewer for this comment. We have included additional literature as highlighted in track changes in our submission.

	caregivers of OVC are likely to be more supportive in different ways that improve the OVC's wellbeing including VL suppression." This seems unexplored. More could be said about this. How do the authors suppose that this is impacting viral suppression? Is it providing more access? Is there a protective mechanism at play. If the introduction had been set up better then this would provide evidence of answering these questions. It is also important for the authors to include information about the implications of the findings	
6	The authors comment that male caregivers are associated with lower viral suppression, but do not provide an explanation or how to better address this. Male caregivers can't be removed. How do the authors suggest they be supported? What evidence is suggested as a cause for this relationship - are we devoting more support for females and leaving males out? More meat is needed here.	Our sincere appreciation to the reviewer for this comment. We have included additional content in the discussion section highlighted in track changes in our submission.
7	The authors state the inability to look at key community and social factors but describe many causes and examine factors at these levels. There are societal and community level factors explored through economic advancement (voluntary savings) and social support. I don't see that PSS is commented on anywhere in the manuscript, which is a big oversight.	Again, we thank the reviewer for this comment. We have included additional literature as highlighted in track changes in our submission.
