# Supplementary material for: Factors associated with viral load suppression among orphans and vulnerable children and adolescents living with HIV in Kenya
Source: PLOS Glob Public Health. 2023 Mar 23;3(3):e0000794. doi: 10.1371/journal.pgph.0000794 (PMC10035747; doi:10.1371/journal.pgph.0000794)
Supplement: Attachment — Submitted filename: Response to Reviewers Comments_Sept 8, 2022_HNJDRB.docx [file pgph.0000794.s001.docx]

| **Response to Editor’s and Reviewers’ Comments**  **Factors associated with viral load suppression among orphans and vulnerable children and adolescents living with HIV in Kenya (Manuscript ID: PGPH-D-22-00954)** |  |
| --- | --- |
|  |  |

September 8th, 2022

Dear Julia Robinson,

Thank you for inviting us to submit a revised manuscript. We are grateful to the editor and reviewers for their insightful comments and suggestions that, we believe, strengthen this manuscript. My team has taken time to work on the manuscript again. Please, allow us to present detailed responses to reviewers’ comments in a table format:

Sincerely,

Brian Onyango.

=========================================================================================================================

**Response to Editor’s Comments**

| **Editor’s Comments on Journal Requirements** | | **Author’s Responses** |
| --- | --- | --- |
| 1 | Please update your online Competing Interests statement. If you have no competing interests to declare, please state: “The authors have declared that no competing interests exist | The statement, “The authors have declared that no competing interests exist,” has been added. |
| 2 | Please amend your detailed online Financial Disclosure statement. This is published with the article. It must therefore be completed in full sentences and contain the exact wording you wish to be published. Please state what role the funders took in the study. If the funders had no role in your study, please state: “The funders had no role in study design, data collection and analysis, decision to publish, or preparation of the manuscript.” | Done. This study did not receive any funding. |
| 3 | In the Funding Information you indicated that no funding was received. Please revise the Funding Information field to reflect funding received. Please ensure that the funders and grant numbers match between the Financial Disclosure field and the Funding Information tab in your submission form. Note that the funders must be provided in the same order in both places as well. | Done. This study did not receive any funding. |
| 4 | Please review your reference list to ensure that it is complete and correct. If you have cited papers that have been retracted, please include the rationale for doing so in the manuscript text or remove these references and replace them with relevant current references. Any changes to the reference list should be mentioned in the rebuttal letter that accompanies your revised manuscript. If you need to cite a retracted article, indicate the article’s retracted status in the References list and also include a citation and full reference for the retraction notice | We carefully reviewed each paper cited using PubMed and noted that none was retracted. For technical reports, we have added the URL from where each can be retrieved. |

**Response to Reviewer 1’s Comments**

| **Reviewer 1’s Comments** | | **Author’s Responses** |
| --- | --- | --- |
| 1 | Manuscript needs significant copy editing. There were many typos and grammatical errors. | The manuscript has been reviewed and significant copy edited to respond to this concern. This can be confirmed from the document shared with track changes. |
| 2 | This reviewer was also concerned by the lack of IRB review. Best practice is always to include IRB review, even for secondary data analysis. This is a non-negotiable requirement when working on PEPFAR (through CDC) awards and represents a basic tenet of Good Clinical Practice in research | The study utilized anonymized secondary programmatic monitoring data extracted from USAID local implementing partner projects of a large PEPFAR funded program in Kenya. No ethical approval was sought. However, administrative written permissions and concurrence to extract data from the partners Child Protection Management Information System databases was obtained from Government of Kenya (GOK) through the Department of Children Services (DCS) and USAID country office through the Health, Population and Nutrition Office and the Agreement Officer Representatives (AORs). |

**Response to Reviewer 2’s Comments**

| **Reviewer 2’s Comments** | | **Author’s Responses** |
| --- | --- | --- |
| 1 | The attempt to characterize factors associated with virologic suppression in OVC-CALHIV is commendable. However, there is need to clarify some aspects of the manuscript in its present form: Overall Objective: the authors need to make clear what outcome is being assessed-is it virologic suppression OR non-suppression? The objective stated in the introduction section is at variance with the line of discussion of results. | This has been addressed. The study aims to identify correlates of optimal viral load suppression among CALHIV in Kenya. The reference levels have also been removed. |
| 2 | In the results section and reflected in Tables 1 and 2: the authors should show and document if there are any differences between those with suppressed vs. unsuppressed viral load across the variables that are listed in the 2 Tables (show results of Chi-square tests or other tests conducted). This will also allow assessment of which variables were appropriate to include in the Model. | The p-values have been included in both Table 1 and 2. |
| 3a | Table 1: the age categories should be reworked to conform with either WHO format (0-9, 10-14, 15-18 OR PEPFAR fine age bands-0-4, 5-9, 10-14, 15-18/19 or 18+). This may modify the impact of age on the primary outcome of interest. | No changes in the results. Fortunately, the variable age group (agr-grp) generated in the dataset was already categorized correctly as 0–4, 5–9, 10–14, 15–17, 18+ according to the PEPFAR categorization. The type with the first age category as 1–4 has been corrected. |
| 3b | -there are non-standard abbreviations used in the Tables-these should be spelt out as footnotes to the Tables respectively | Abbreviations provided as table footnotes. |
| 4 | Missing Data: Can the authors explain how they handled missing data in the course of analysis of program level data? were there any sensitivity analyses conducted to handle this limitation? OR were there no missing data elements for any of the variables extracted? | Missing data was not imputed. The data analyzed in this study is part of routine Health records. Data for about 65,816 CALHIV was extracted of which 7,918 are in urban counties and 57,898 in rural counties. After removal or rows of data with missing values, a total sample size of N = 31291 was retained for data analysis. This was considered large enough for complete case analysis. The analysis model applied was the generalized mixed effects model in R (GLMER). |
| 5 | Abstract: The aim stated in the abstract is to characterize SUB-OPTIMAL virologic suppression as opposed to VLS<1000c/ml stated in the main text of the manuscript-please harmonize. Additionally, please delete the "ref level" in the body of the abstract and just include only the aOR's and 95% CIs. Endeavor to define all variables fully in the Methods section and reserve the abstract for key findings only | This has been addressed. The study aims to identify correlates of optimal viral load suppression among CALHIV in Kenya. The reference levels have also been removed. |
